# Infected Abdominal Aortic Aneurysm with* Helicobacter cinaedi*

**DOI:** 10.1155/2016/1396568

**Published:** 2016-01-17

**Authors:** Kazuhiro Nishida, Takamasa Iwasawa, Atsushi Tamura, Alan T. Lefor

**Affiliations:** ^1^Department of Surgery, Yokosuka General Hospital, Uwamachi, Kanagawa 238-8567, Japan; ^2^Department of Cardiovascular Internal Medicine, Yokosuka General Hospital, Uwamachi, Kanagawa 238-8567, Japan; ^3^Department of Cardiovascular Surgery, Yokosuka General Hospital, Uwamachi, Kanagawa 238-8567, Japan; ^4^Department of Surgery, Jichi Medical University, Tochigi, Japan

## Abstract

*Helicobacter cinaedi* is a rare human pathogen which has various clinical manifestations such as cellulitis, bacteremia, arthritis, meningitis, and infectious endocarditis. We report an abdominal aortic aneurysm infected with* Helicobacter cinaedi*, treated successfully with surgical repair and long-term antimicrobial therapy.

## 1. Introduction

*Helicobacter cinaedi* (*H. cinaedi*) was first reported in 1985 from the rectal swab of a homosexual man with proctocolitis [1]. This organism is usually isolated from immunocompromised patients, such as HIV infected patients, but recent data shows that immunocompetent hosts can also be infected [2, 3]. A multicenter analysis in Japan found that* H. cinaedi* was isolated from 0.22% of positive blood cultures [4]. This organism has various clinical manifestations, including cellulitis, bacteremia, arthritis, infectious endocarditis, prosthetic graft infection, and infected aneurysms [3–7]. We describe a patient with* H. cinaedi* infected abdominal aortic aneurysm.

## 2. Case Presentation

A previously healthy 64-year-old man presented with progressive low back pain over two days. His temperature was 36.4°C and there was no abdominal or low back tenderness on physical examination. There was no humoral immunity deficit and HIV antigen/antibody test was negative. A contrast enhanced computed tomography scan showed an infrarenal aortic aneurysm with an eccentric wall that extended to the left common iliac artery, measuring 55 mm in diameter. The following day he had a fever and an infected abdominal aortic aneurysm was suspected. Three sets of blood cultures were obtained and meropenem and vancomycin begun. Due to sepsis induced respiratory failure and acute kidney injury, he was transferred to the intensive care unit and treated with mechanical ventilation and hemodialysis. One set of blood culture turned positive and* H. cinaedi* was identified by 16S rRNA sequence analysis with 99% similarity and amplification of* gyr* B gene which is specific to* H. cinaedi*. Since the aneurysm had rapidly enlarged to 70 mm, surgical repair with a prosthetic graft was performed on hospital day 27. Pathological analysis of specimen revealed atherosclerosis and infiltration of neutrophils which is consistent with infected aneurysm. He did well postoperatively. After a total of eight weeks of antibiotic treatment, he was discharged on postoperative day 34.

## 3. Discussion

*H. cinaedi* is a Gram negative spiral rod which colonizes the gastrointestinal tract of various animals such as hamsters and rhesus monkeys [8]. Contact with animals is thought to be a risk factor for infection, which our patient denied. Diagnosis can be difficult because it rarely grows in traditional culture media. Identification of the organism is based on 16S ribosomal RNA sequence analysis [4, 5]. Optimal duration of therapy is still unknown. Uçkay et al. recommend prolonged antibiotic therapy because of the potential for recurrence [5]. In the present patient, the isolate was sensitive to penicillins, cephalosporins, and minocycline but resistant to macrolides, fluoroquinolones, and vancomycin. Postoperative minocycline for two weeks and cefazolin for four weeks were administered. Dubois et al. reported 44 cases of infected abdominal aortic aneurysms.* In situ* reconstruction was more often performed than extra-anatomic reconstruction (37 versus six), and in-hospital mortality was low (18.9% versus 50%), but three patients had recurrent infections [9]. Kakuta et al. reported three patients with* H. cinaedi* infected abdominal aortic aneurysms treated with* in situ* reconstruction, and all patients survived [7]. In this patient,* in situ* reconstruction was performed, without evidence of recurrence after two-year follow-up.

## 4. Conclusion

A patient with a* H. cinaedi* infected abdominal aortic aneurysm was treated successfully with surgical repair and long-term antimicrobial therapy.
